# Sylvian fissure meningioma: A case report and systematic review of clinical and surgical insights

**DOI:** 10.1016/j.bas.2025.104310

**Published:** 2025-07-07

**Authors:** Mohammed A. Azab, Khalid Sarhan, Oday Atallah, Ahmed Sabra, Brahim Kammoun, Ahmed Hazim, Sara Hamed, Mohsen Nabih Shama

**Affiliations:** a Department of Endovascular Surgical Neurology, OBH-Brookdale University Hospital, Brooklyn, NY, USA; b Faculty of Medicine, Mansoura University, Mansoura, Egypt; c Department of Neurosurgery, Carl Von Ossietzky University, Oldenburg, Germany; d W.M. Keck Center for Collaborative Neuroscience, Rutgers, The State University of New Jersey, Piscataway, NJ, USA; e Department of Neurosurgery – Habib Bourguiba University Hospital–Sfax (Tunisia), Tunisia; f Department of Neurosurgery, Cairo University Hospital, Cairo, Egypt; g Department of Emergency Medicine, KAMC, KSA, Saudi Arabia

**Keywords:** Sylvian fissure, Meningioma, Dural attachment, MCA

## Abstract

**Background:**

Meningothelial cells within the arachnoid of the Sylvian fissure give rise to a rare subtype of non-dural based tumors known as Sylvian fissure meningiomas (SFMs). The clinical presentations and management of these lesions need to be further explained.

**Methods:**

We followed the PRISMA statement guidelines when reporting this systematic review and meta-analysis. We conducted a literature search through PubMed, Embase, and Web of Science.

**Databases:**

We performed a quantitative summary of all included studies. We also describe an additional case report of a grade II Sylvian fissure meningioma (SFM).

**Results:**

A total of 40 patients with sylvian fissure meningioma were analyzed. The mean (range) age was 23.4 (1.5–73) years, 24 were adults and 16 were children with a female-to-male ratio of 15:25. Most patients with Sylvian fissure meningioma (SFM) presented with seizures 28 (70 %). According to the WHO histological grading of meningiomas, a total of 32 (80 %) tumors were diagnosed as grade I meningiomas and 8 (20 %) were WHO grade II. Pterional craniotomy was the most frequently employed approach, 21 (67.7 %). In 35 studies reporting surgical resection outcomes, gross total resection was achieved in 25 (71.4 %) patients, whereas subtotal resection was necessary in 10 (28.6 %) patients. Postoperative complications were present in 10 out of 40 patients.

**Conclusion:**

WHO grade II SFMs are a rare subset of supratentorial meningiomas. The most common symptom is seizure. Imaging reveals no dural tail like other typical meningioma characteristics. The cornerstone of therapy is surgery. It is necessary to assess whether such a location has a positive or negative predictive value in larger case series cohorts.

## Highlights

• Meningothelial cells within the arachnoid of the Sylvian fissure give rise to a rare subtype of non-dural based tumors known as (SFMs).
• Sylvian fissure meningiomas are rare in the literature, the total number of case reports is 42 cases.
• Sylvian fissure meningiomas show unique radiological features, such as an intra axial mass devoid of dural connection.
• Seizures are more frequent in SFMs.
• A significant predictor of inadequate resection is the close relation to the MCA vessels.

## 1 Introduction

Meningiomas arise from the arachnoid cap cells, which are usually located close to the granulations, arachnoid, and dura mater explaining why these lesions usually have a dural attachment (Evans et al., 2006; Park et al., 2005; Perry et al., 2007). These cells are also located in the choroid plexus and tela choroidea, therefore, certain meningiomas could arise without dural attachment (Chiocca et al., 1994). Intraventricular, pineal area, and fourth ventricular meningiomas are typical examples of meningiomas without dural attachment (Chiocca et al., 1994). The pediatric population is the most frequent to harbor meningiomas without dural attachment, and they are frequently located in the infratentorial compartment. We report a case of meningioma (World Health Organization [WHO] grade II) located in the Sylvian fissure and examine the literature to outline the clinical, radiological, pathological, and surgical characteristics of Sylvian fissure meningiomas (SFMs).

## 2 Case illustration

**Fig. 1:**
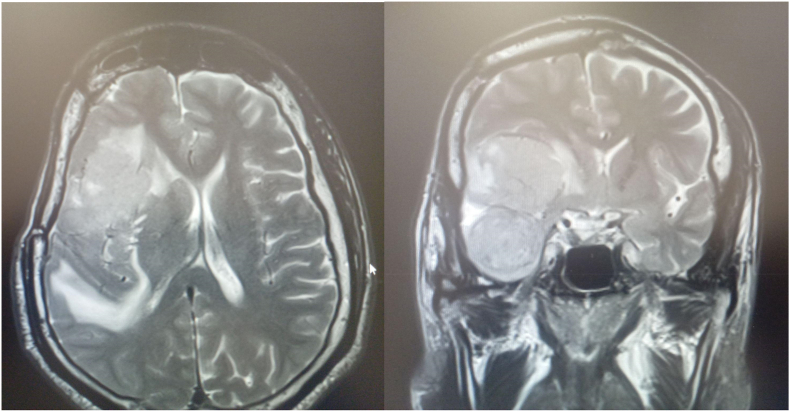
MRI brain T2 sequence showing a right sylvian fissure meningioma with surrounding perifocal edema.

A 55-year-old male visited our hospital with a 6-month history of refractory seizures that increased in frequency over the last month. No physical abnormalities or neurological deficits were observed on admission. The laboratory tests and other systemic evaluations were normal. Magnetic resonance imaging (MRI) revealed a 5-cm, well-circumscribed mass that was isointense with the brain cortex on both the T1-and T2-weighted images with a marked peritumoral edema Fig. 1. It was believed to be located in the anterior and middle Sylvian region occupying a part of the insular region and there was no dural attachment. Branches of the middle cerebral artery (MCA) were attached to the deep part of the tumor. Enhancement of the tumor was homogeneous and avid.

A left pterional craniotomy was then performed. The tumor was not exposed until the sylvian fissure was dissected, and a firm tumor was found buried in the sylvian fissure. The M2 ran over the tumor's surface and was splayed out, which appeared to be a part of the tumor capsule. The tumor had no dural attachment and was very firmly attached to the M2. In addition, it was very difficult to separate the M2 from the tumor.

**Fig. 2:**
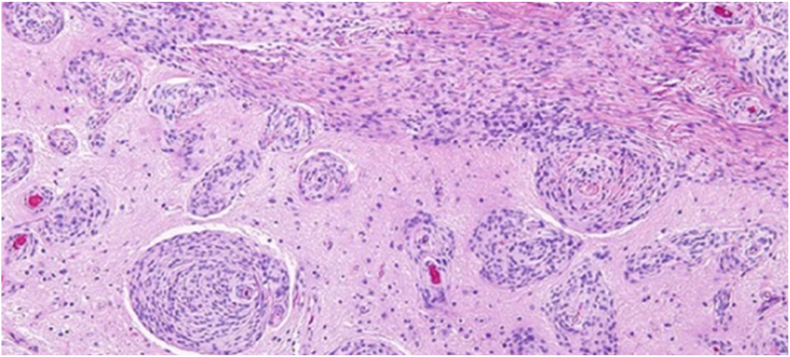
H&E-stained section showing evident brain invasion.

The histological features were consistent with an atypical meningioma, WHO grade II Fig. 2. There were areas of hypercellularity and necrosis, with a low-proliferative index. The postoperative course was uneventful. The patient reported a gradual improvement in his seizure frequency over the year following surgery. A follow-up MRI was done over 1 and 2 years that revealed a stable residual.

## 3 Methods

We followed the PRISMA statement guidelines when reporting this systematic review and meta-analysis. We established the methods before conducting the review, and no significant deviations from the protocol were noted.

### 3.1 Search strategy

We conducted a literature search through PubMed, Embase, and Web of Science databases. The following keywords were used: “Sylvian fissure”, “meningeal tumors”, “meningioma”. Search terms were derived from available reviews on sylvian fissure meningiomas, including key references from studies.

### 3.2 Screening and data extraction

After removing the duplicates, three reviewers used Rayyan software (Cambridge, Massachusetts, USA) to evaluate the resulting studies for inclusion and/or removal based on abstract and title. For the abstracts involved, the senior author determined the inclusion and exclusion criteria. The most recent search was conducted in March 2025.

Inclusion criteria included: studies explicitly describing Sylvian fissure meningiomas in case reports or case series. Conference reports, animal studies, reviews, and studies where other intracranial meningiomas without specific data on Sylvian fissure meningiomas were among the exclusion criteria. We performed data extraction on a spreadsheet including study ID, patient demographics, clinical presentation, surgical details, extent of resection, postoperative complications, and recurrence rates.

### 3.3 Statistical analysis

We performed the meta-analysis using Microsoft Excel and Jamovi software. We performed a quantitative summary of all included. The findings were summarized in figures and tables to highlight trends in clinical presentation, tumor characteristics, surgical approach, and outcomes Table 1, Table 2

## 4 Results

### 4.1 Summary of the search results:

**Fig. 3:**
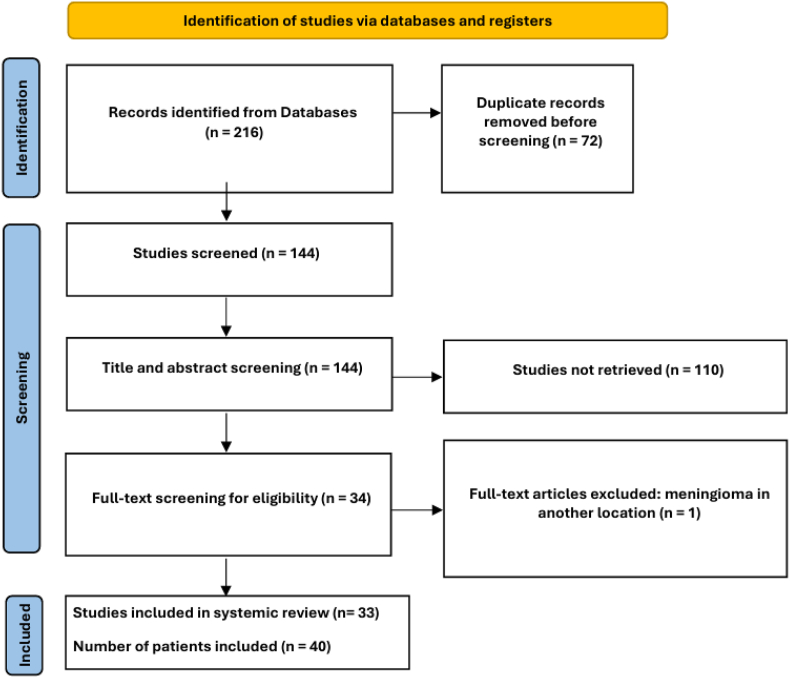
PRISMA flow chart of the included studies and number of patients.

Four databases were utilized to identify 144 articles after removing duplicates. Of the articles, 110 were excluded based on ineligibility determined by screening titles and abstracts. Two investigators evaluated the entire contents of the remaining 34 articles and ultimately identified 33 articles including 40 patients eligible for the study Fig. 3.

### 4.2 Epidemiology and clinical presentations

**Fig. 4:**
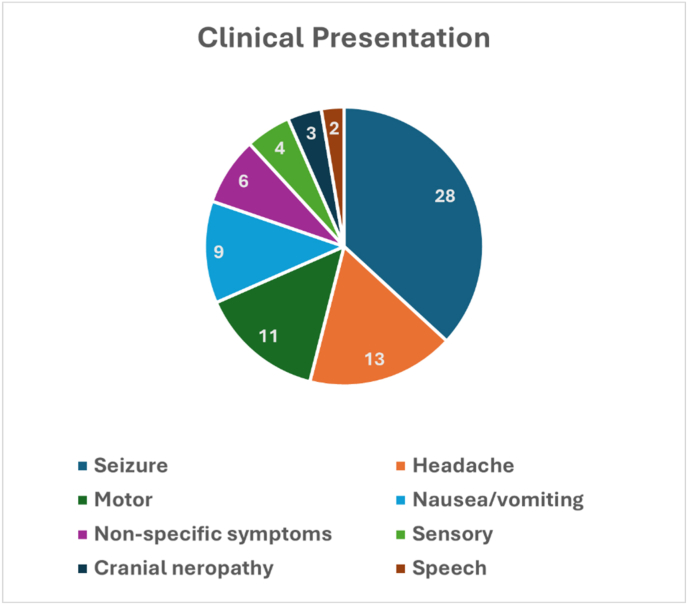
Clinical Presentation of patients with SFMs.

A total of 40 patients Table 1 with SFMs were analyzed. The mean (range) age was 23.4 (1.5–73) years. A total of 24 patients were adults Table 2 and 16 were children Table 3 with a female-to-male ratio of 15:25. Most patients with sylvian fissure meningioma presented with seizures 28 (70 %), headaches 13 (32.5 %), and nausea/vomiting 9 (22.5 %). Other symptoms included motor deficits in 11 (27.5 %) patients, sensory deficits in 4 (10 %) patients, cranial neuropathy in 3 (7.5 %) patients, and speech in 2 (5 %) patients. A total of 6 (15 %) patients presented with non-specific symptoms. No patients were diagnosed incidentally Fig. 4.

**Table 1:** Summary of the studies included.

Variable	No. (%)
No. of studies	33
No. of unique patients	40
Mean age (range)	23.4 (1.5–73)
Sex (female), n (%)	15 (37.5 %)
**WHO histological classification, n = 40**	
I	32 (80 %)
II	8 (20 %)
**Tumor location in sylvian fissure, n = 30**	
Ant	7 (23.3 %)
Middle	5 (16.7 %)
Posterior	3 (10 %)
Ant and middle	10 (33.3 %)
Middle and posterior	2 (6.7 %)
All	3 (10 %)
**Tumor size, n = 19**	
Mean (SD)	4.18 (1.83)
**Vascular relation, n = 36**	
Yes	27 (75 %)
No	9 (25 %)
**Surgical resection, n = 35**	
Gross total resection	25 (71.4 %)
Subtotal resection	10 (28.6 %)
**Follow up duration, n = 26**	
Mean (range)	3.35 (0.13–13)

**Table 2:** Summary of the studies of SFMs among adult populations.

Study	Age/Sex	C/P	Surgical approach	Resection	WHO grade	Complications	F/U (Y)
**Zhang et al** (Zhang and Wang, 2017)	42/M	Headache	Temporal craniotomy	GTR	1	None	1
**Barcia-Goyanes et al** (BARCIA-GOYANES and CALVO-GARRA, 1953)	20/F	Seizures	NR	NR	1	NR	NR
**Graziani et al** (Graziani et al., 1992b)	19/M	Headache, nausea, motor symptoms	NR	GTR	1	None	NR
**Cushing et al** (Cushing and Eisenhardt)	18/M	Seizures	Pterional	STR	1	None	5
	48/F	Seizures	Pterional	NM	1	Death	–
**Chang et al** (Chang et al., 2005)	35/M	seizures	Pterional	STR	1	None	NR
**Cai et al** (Cai et al., 2020)	30/M	seizures	Temporal craniotomy	GTR	2	hemiplegia	1.5
**Yamagishi et al** (Yamagishi et al., 2019)	32/M	Headache	Pterional	GTR	1	None	NR
**Eghwrudjakpor et al** (Eghwrudjakpor and Mori, 2006b)	73/F	Fatigue	Temporoparietal craniotomy	GTR	1	Progressive hydrocephalus- subgaleal fluid collection	NR
**Brogna et al** (Brogna et al., 2018)	32/M	Headache, nausea, vomiting	Pterional	GTR	2	None	3
**McIver et al** (McIver et al., 2005b)	23/M	Seizures	Pterional	GTR	2	None	1.5
**Chiocca et al** (Chiocca et al., 1994)	26/F	Seizures	Parietal Craniotomy	GTR	1	None	NR
**Cecchi t al** (Cecchi et al., 2009)	23/M	Headache	Pterional	STR	2	transient speech problem	2
**Aras et al** (Aras et al., 2013)	28/M	Seizures	Temporal craniotomy	STR	1	None	5
**Ma et al** (Ma et al., 2012b)	53/M	Seizures	Pterional	STR	2	None	2
**Wang et al** (Wang et al., 2022)	26/M	Seizures	NR	Pterional	1	Anomic aphasia and weakness in his left limb	1
**Saito et al** (Saito et al., 1979)	31/F	Seizures	Pterional	GTR	1	None	NR
**Tsuchida et al** (Tsuchida et al., 1981)	46/M	Headache	NR	GTR	1	None	NR
**Okamoto et al** (Okamoto et al., 1985)	27/F	Headache, nausea, vomiting	Pterional	STR	1	Hemiparesis	6
	35/F	Seizure, sensory symptoms	Temporoparietal craniotomy	GTR	1	Dysarthria	NR
**Hirao et al** (Hirao et al., 1986b)	34/F	Seizures	NR	GTR	1	NR	NR
**Matsumoto et al.** (Matsumoto et al., 1995)	62/F	Headache, seizures	Pterional	GTR	1	None	NR

GTR gross total resection, NR not reported, STR subtotal resection.

**Table 3:** Summary of the studies of SFMs among pediatric populations.

Study	Age/Sex	C/P	Surgical approach	Resection	WHO grade	Complications	F/U (Y)
**Hong et al** (Hong et al., 2019)	1.5/F	Headache, nausea, vomiting	Temporal craniotomy	GTR	2	None	1
**Mitsuyama et al** (Mitsuyama et al., 2000)	1.8/M	Seizures	Pterional	GTR	NR	NR	NR
**Cho et al** (Cho et al., 1990)	2/M	Seizures, motor symptoms	Frontoparietal craniotomy	GTR	1	None	NR
**Cooper et al** (Cooper et al., 1997)	4/M	Headache, nausea, vomiting	NR	GTR	1	left hemiparesis	1
**Silbergeld et al.** (Silbergeld et al., 1988a)	4/F	Seizures, nausea, vomiting	Temporal craniotomy	GTR	1	None	1
**Amirjamshidi et al** (Amirjamshidi et al., 2019)	5/F	Headache, seizures	Pterional	GTR	I	None	5
	7/F	Headache, nausea, vomiting	Pterional	GTR	I	None	13
	7/M	Headache, seizures	Pterional	GTR	I	None	2
**Samson et al** (Samson Sujit Kumar and Rajshekhar, 2009)	6/M	Seizures	Temporo-parietal craniotomy	GTR	1	None	4
**Donovan** (Donovan and Thavapalan, 2016)	7/M	Seizures	Pterional	STR	II	Cerebral edema- expressive aphasia	7
	11/M	Seizures	Pterional	GTR	I	None	10
	16/F	Seizures	Pterional	STR	I	None	5
**Doty et al** (Doty et al., 1987)	8/M	Seizures	NR	NR	1	None	3
**Fukushima** (Fukushima et al., 2014)	10/M	Seizures	Pterional	GTR	I	Basal ganglia ischemia	1
**Kaplan et al** (Kaplan et al., 2002b)	11/F	Seizures	Pterional	NR	2	None	NR
**Aras et al** (Aras et al., 2013)	15/M	Seizures	Pterional	STR	1	None	3

GTR gross total resection, NR not reported, STR subtotal resection.

### 4.3 Tumor characteristics

**Fig. 5:**
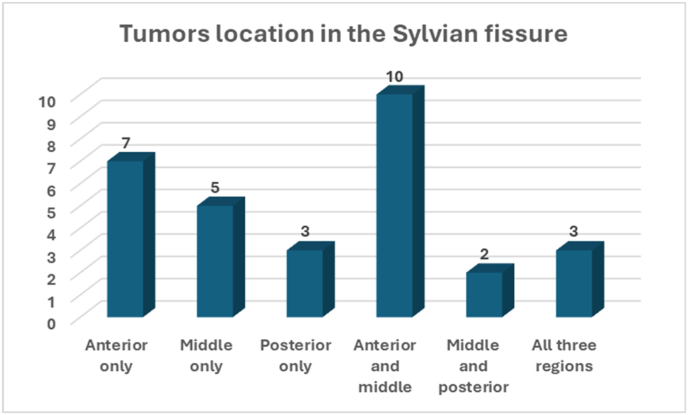
Tumors location within the Sylvian fissure.

The tumors were localized in different regions of the Sylvian fissure (n = 30), with 7 (23.3 %) tumors being in the anterior region only, 5 (16.7 %) in the middle region only, and 3 (10 %) in the posterior region only. Additionally, 10 (33.3 %) tumors were extending in both anterior and middle regions of the Sylvian fissure, 2 (6.7 %) tumors were in the middle and posterior regions, and 3 (10 %) tumors spanning along all three regions (anterior, middle, and posterior) Fig. 5. The mean (SD) tumor size for 19 patients was 4.18 (1.83). A total of 27 tumors are closely related to the MCA and 9 have no relations. According to the WHO histological grading of meningiomas, a total of 32 (80 %) tumors were diagnosed as grade I meningiomas and 8 (20 %) were WHO grade II Table 1.

### 4.4 Surgical approaches and outcomes

**Fig. 6:**
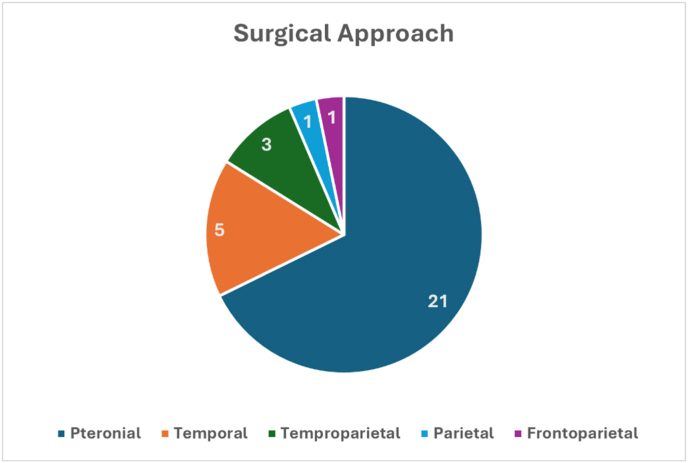
Surgical approaches for patients with SFMs.

**Fig. 7:**
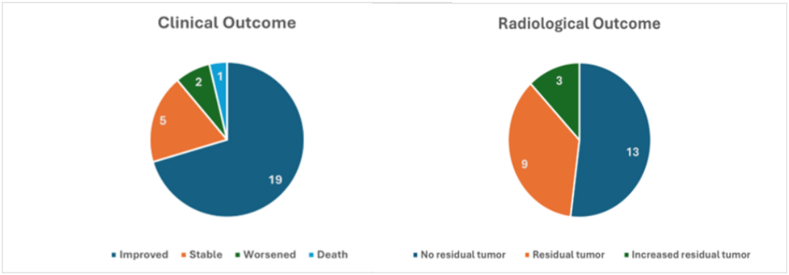
Postoperative clinical and radiological outcomes of SFMs.

In 31 studies, the surgical approaches for management of SFMs are reported. Pterional craniotomy was the most frequently employed approach, 21 (67.7 %). Other approaches included temporal craniotomy 5 (16.1 %), temporoparietal 3 (9.7 %), parietal 1 (3.2 %), and frontoparietal 1 (3.2 %) Fig. 6. In 35 studies reporting surgical resection outcomes, gross total resection was achieved in 25 (71.4 %) patients, whereas subtotal resection was necessary in 10 (28.6 %) patients Table 1. Postoperative complications were present in 10 out of 40 patients including, expressive aphasia, transient speech problems, dysarthria, hemiparesis, hemiplegia, cerebral edema, hydrocephalus, basal ganglia ischemia, regrowth, and death (1 patient). Adjuvant therapies included gamma knife radiosurgery in 2 patients and 1 patient received radiation therapy (6000 cGy). Out of 27 studies reporting postoperative clinical outcomes and 25 studies reporting postoperative radiological outcomes, most patients 19 (70.4 %) had improved postoperative clinical outcomes, 5 (18.5 %) were stable, 2 (7.4 %) worsened, and 1 (3.7 %) died. No residual tumor was found in 13 (52 %) patients, 9 (36 %) still had residual tumor, while only 3 (12 %) patients had increased residual tumor size Fig. 7. The mean (range) follow-up duration was 3.35 (0.13–13) years Table 1. Tumor recurrence occurred in 4 patients, 3 of which underwent a second surgery. One patient died after the second surgery and another patient had no recurrence after 3 years of follow-up.

## 5 Discussion

Meningiomas are the second most common (approximately 25 %) intracranial tumor following glioma (Silbergeld et al., 1988a). They typically affect adults the most, with a median age of diagnosis being 65 years (Cushing and Eisenhardt, 1969). The pediatric patients, particularly in the 10- to 15-year-old age group (62 %) are also affected and around 40 % of these individuals typically have neurofibromatosis (Cushing and Eisenhardt, 1969; Mori et al., 1994).

The majority of meningiomas are benign, slowly growing lesions arising from the arachnoid cap cells with a dural attachment. The arachnoid cap cells may be present in the pia mater of the deep Sylvian fissure and the Virchow-Robin spaces close to the MCA and its branches. Those are included in a broad category termed (meningioma without a dural attachment) as reported by Cushing (Cushing and Eisenhardt, 1969; Mori et al., 1994). They are classified into three major groups: intraventricular, subcortical, and deep Sylvian (Cushing and Eisenhardt, 1969). Intraventricular meningiomas are well-studied, while the SFMs are poorly exposed (Mori et al., 1994; Bhatoe et al., 2006). Excluding intraventricular meningiomas, few case reports described other meningiomas lacking dural attachment especially the SFMs, and they primarily affected younger male generations (average age 26. 5 years) (Cooper et al., 1997; Eghwrudjakpor and Mori, 2006a; Graziani et al., 1992a; Hirao et al., 1986a; Kaplan et al., 2002a; Matsumoto et al., 1995; McIver et al., 2005a). It is quite challenging to differentiate them from a high-grade glioma, a metastatic brain tumor, lymphoma, or granulomas. We reported an unusual (WHO grade II) deep Sylvian meningioma, and we also highlighted the relevant literature to uncover a comprehensive description of this rare meningioma subtype.

### 5.1 Demographics

Typical meningiomas are always discovered at the age 55, and as people age, their incidence rises, with women experiencing higher morbidity than men (Wiemels et al., 2010). According to a retrospective report of 38 patients with SFMs recorded between 1938 and 2019, the average age is 22.5 ± 17.4 years, with 25 patients being men (65.8 %) and 13 being women (34.2 %) (Cai et al., 2020). In our updated analysis, the mean age was 23.4 (1.5–73), with 25 patients being men (62.5 %) and 15 being women (37.5 %) which is similar to the old analysis. They make up 0.5–5 % of pediatric central nervous system (CNS) tumors, making them comparatively uncommon in the pediatric population (Desrosiers-Battu et al., 2025). In our cohort, SFMs were reported to affect the pediatric population. Accordingly, we found 10 case reports with available data about SFMs in pediatrics (Amirjamshidi et al., 2019; Fukushima et al., 2014; Kaplan et al., 2002b; Samson Sujit Kumar and Rajshekhar, 2009; Aras et al., 2013).

### 5.2 WHO classification

About 16.9 % of cases are (WHO grade II), while the majority of meningiomas are WHO grade I (Ostrom et al., 2016). According to the 2016 WHO classification of central nervous system tumors, the presence of brain invasion—which was added to the histological criteria—can help diagnose atypical meningiomas alone (Louis et al., 2016). In a previously published literature review, psammomatous (8 cases, 21.1 %), transitional (7 cases, 18.4 %), meningothelial (7 cases, 18.4 %), fibroblastic (7 cases, 18.4 %), atypical (4 cases, 10.5 %), chordoid (1 case, 2.6 %), sclerosing (1 case, 2.6 %), lymphoplasmacytic (1 case, 2.6 %), and malignant (1 case, 2.6 %) were the histological diagnoses for the reported cases of SFMs (Cai et al., 2020). In our analysis, 80 % were grade I, while only 20 % were grade II. Our case showed parenchymal invasion with perifocal edema, and it was proven to be grade II.

### 5.3 Anatomico-radiological features

Dura mater attachment (dural tail sign) and extra-axial location are typically the primary radiological features of meningiomas (Saloner et al., 2010). On the other hand, sylvian fissure meningiomas show unique radiological features, such as an intra axial mass devoid of dural connections. The reported 40 cases of SFMs frequently showed as hypointense or isointense on T1-and T2-weighted MRI, with homogeneous or heterogeneous enhancement.

Since SFMs are uncommon, it's critical to distinguish them from sphenoid wing meningiomas. In contrast to the SFMs, which grow between the MCA branches and lack dural attachment and hyperostosis, sphenoid wing meningiomas are attached to the dura covering the sphenoid wings and are typically linked to hyperostosis. MCA and its branches are more prone to sustain damage during resection of SFMs due to their close relation to these lesions (Chang et al., 2005). When the surrounding arachnoid plane is preserved, surgical resection can be performed without endangering the nearby cortex. Furthermore, meningiomas can be adherent to the adventitia of cerebral arteries or even fully encase an artery, making it difficult to differentiate the tumor from native blood vessels (Brokinkel et al., 2017). In this review, about 75 % of the lesions were enclosed or near the MCA. Only 5 patients had ischemic neurological complications related to MCA manipulation or injury. A rare side effect of supratentorial meningioma excision is cerebral vasospasm (Pickles et al., 2024). Sharp microdissection techniques are frequently employed; however, vessel manipulation is often inevitable. Cranial MRI and DSA made it easier for early clinical identification of lateralizing symptoms linked to focal cerebral ischemia. Significant morbidity and mortality should be avoided with prompt management.

### 5.4 Clinical presentations

Englot et al. found that seizures are more frequent in SFMs than other supratentorial meningiomas (Englot et al., 2016). In a review by Cio et al., seizure was the major presenting symptom of SFMs (65.8 %) 25 patients (Cai et al., 2020). In our analysis, seizures also were the most common presenting symptom of 28 patients (70 %). Certain factors could increase the risk of postoperative seizures in cases of SFMs, which are common attributable factors associated with other meningiomas. Peritumoral edema is an established factor that could increase the risk of seizures (Teske et al., 2024). The close location to the temporal lobe is to be blamed for increasing incidence of seizures. We observed a strong correlation between meningiomas located in the anterior and middle sylvian regions and the incidence of preoperative seizures. Two-thirds of patients with refractory epilepsy evaluated for possible surgery had temporal lobe seizures, making the temporal lobes the most common site of origin for focal seizures (Blair, 2012). In our illustrative case, the presenting symptom was an associated refractory seizure.

### 5.5 Surgical management

For surgically accessible meningiomas, the preferred method of treatment is total tumor excision (Simpson grade I), which improves prognosis and prevents recurrence (Aizer et al., 2015). The lack of dural attachment is favorable in terms of total surgical excision. However, a significant predictor of inadequate resection has been the close relation to the MCA vessels. For proper surgical planning, preoperative vascular imaging may be useful in defining the vascular anatomy. In a previous literature review, 22 patients (57.9 %) had gross complete resection on the first attempt and the relapse-free status was (92.3 %, 12/13 patients; and other patients had no follow-up data) even after several years (Cai et al., 2020). In our review, 25 (71.4 %) underwent gross total resection, while only 10 (28.6 %) underwent subtotal resection. The primary treatment for meningiomas is complete surgical resection including the dural tail, which is crucial for proper disease control. Complete resection is sometimes challenging and depends upon the tumor's size and anatomical location. To determine the extent of resection (EOR), the Simpson grading system is frequently employed. Although EOR was evaluated as a predictor of both overall and progression-free survival (OS and PFS) in a number of studies, none of them precisely addressed the preoperative factors that influence EOR.

Tumor recurrence occurred in 4 patients, 3 of which underwent a second surgery, and the follow up period ranged from 0.13 to 13 years. No residual tumor was found in 13 (52 %) patients, 9 (36 %) still had residual tumor, while only 3 (12 %) patients had increased residual tumor size. The presence of residuals was related in most cases due to possible involvement of the MCA and its branches within the tumor. Gamma knife radiosurgery was used for treating a residual lesion in a single case (Ma et al., 2012a). For certain meningiomas, radiation therapy has emerged as a first-line treatment, especially for small meningiomas in challenging anatomical sites (McIver et al., 2005a). Surgery and SRS combination therapies are also becoming more popular; however, there are still debates about the best timing and modality for radiation therapy. Sheehan et al. conducted a significant study that compared active surveillance and SRS alone in cases of asymptomatic incidental meningiomas (Sheehan et al., 2022). They reported that SRS provides better radiologic tumor control than active surveillance with far less complications. Gamma-knife radiosurgery may be suggested for patients who are at risk of severe complications related to surgery (Cai et al., 2020; Silbergeld et al., 1988b; Rogers et al., 2018).

### 5.6 Limitations

Our systematic review and pooled analysis have several limitations. First, the sample size of available case reports is small because the Sylvian fissure is a very rare location to harbor meningioma with only 40 cases reported in the literature. A more thorough analysis will be possible with larger samples. Moreover, due to inadequate available data and short follow-ups, we were not able to properly determine whether survival analysis or progression free survival. The follow up period was short in most studies. Long term longitudinal studies, and large sized case series are required to properly evaluate the outcome of patients with SFMs and to determine the best surgical management guidelines for this rare subtype.

## 6 Conclusion

SFMs are a unique category of meningiomas with distinct radiological and clinical features. The absence of major common features for meningioma confounds the diagnosis with intra axial lesions. It is necessary to assess whether such a location has a positive or negative predictive value in larger case series cohorts.

## Informed consent

Verbal and written consent was given by the patient.

## Author contributions

Conception and design: M.A. and S.A. acquisition of data and analysis: M.A and K.S. writing – original draft preparation: M.A., S.A; and A.H writing – reviewing and/or editing of manuscript: M.A. and O.A. All authors have read and agreed to the published version of the manuscript.

## Consent for publication

We certify that the material has not been published anywhere.

## Ethical approval

The study protocol was reviewed by the Regional Ethical Committee of Cairo University Hospital as it classifies as a quality improvement study, which only requires permission from the local hospital.

## Funding

No funding was received for this research.

## Declaration of competing interest

The authors have no Conflict of interest and financial support to disclose.

## Abbreviations:

GTR: gross total resection
MCA: middle cerebral artery
NR: not reported
SFMs: Sylvian Fissure Meningiomas
STR: subtotal resection
